# Divergent Bird Diversity Patterns Among Four Airports in the Same Bioregion: Assessing Local‐Scale Drivers of Bird Community Assembly

**DOI:** 10.1002/ece3.71772

**Published:** 2025-07-13

**Authors:** Wan Chen, Caiqian Sun, Yuanyuan Cai, Hang Zhang, Weiya Qian, Peng Li, Xinglong Huang, Qing Chang, Chaochao Hu

**Affiliations:** ^1^ College of Environment and Ecology Jiangsu Open University (The City Vocational College of Jiangsu) Nanjing Jiangsu China; ^2^ School of Life Science Nanjing Normal University Nanjing Jiangsu China; ^3^ Shanghai International Airport co., Ltd. Pudong International Airport Shanghai China; ^4^ Shanghai Hongqiao International Airport Shanghai China; ^5^ Analytical and Testing Center Nanjing Normal University Nanjing Jiangsu China

**Keywords:** airport ecology, bird diversity, bird strike, Yangtze River delta

## Abstract

The rapid expansion of global aviation has intensified conflicts between aircraft and wildlife, particularly bird strikes, which pose significant safety risks and economic losses. Research on airport bird communities have been extensively conducted at both regional and fine scales; however, studies at the local scale remain relatively limited. This study focuses on four airports in China's Lower Yangtze River Plain. From November 2018 to October 2019, point‐count surveys were conducted across four habitat types (farmland, forest, wetland, and residential areas) at each airport. We recorded 147 bird species across airports, consisting of 50 permanent residents and 96 migrants, with 43 species found to be shared among four airports. Despite shared bioregional characteristics, we found significant variations in bird species richness (*H* = 61.25, df = 3, *p* < 0.01), abundance (*H* = 30.86, df = 3, *p* < 0.01), and Shannon‐Wiener index (*H* = 50.49, df = 3, *p* < 0.01) across the four airports. While Nanjing Lukou International Airport recorded markedly higher species richness (125 species; *p* < 0.01 based on post hoc tests), the other three airports formed a distinct group with consistently lower diversity levels. This study revealed the impact of seasonal shifts and habitat variations on bird community dynamics, with bird diversity and composition fluctuating significantly between seasons and across different habitat types. Future research should expand the geographic scope of studies and assess the effectiveness of different management strategies in reducing bird strike. Integrating ecological considerations into airport safety protocols is essential for reducing bird strike hazards while conserving local biodiversity.

## Graphical abstract

A total of 147 bird species was recorded across airports in the Lower Yangtze River Plain. Bird diversity in airport environments varied significantly among different habitat types. Wetlands and farmlands are more likely to attract bird aggregations than other habitat types.

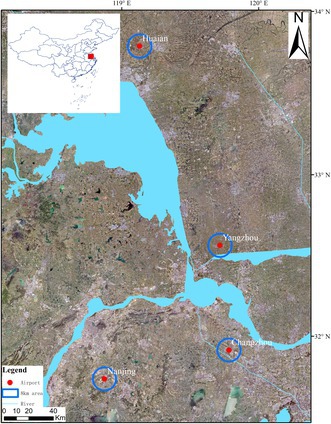

## 1 Introduction

The rapid expansion of the aviation industry has intensified conflicts between wildlife and aircraft, particularly bird strikes, which pose significant risks to aviation safety and result in substantial economic losses globally (DeVault et al. 2016; Jeffery and Buschke 2019; Viljoen and Bouwman 2016). This risk is exacerbated by the tendency of certain bird species to congregate in airport environments, where simplified ecosystems and reduced interspecific competition can enhance their foraging efficiency (Soldatini et al. 2010). Wildlife hazard management, particularly bird population control, has become a critical operational priority for airport authorities worldwide (Blackwell et al. 2019). Understanding the factors driving bird community composition in these areas, such as habitat types, food availability, and landscape structure, is critical for mitigating bird strike risks (Alquezar et al. 2020; Conkling et al. 2018; Yuan et al. 2023).

Bird diversity in airport areas is typically assessed through species richness and patterns of spatial distribution across habitats or temporal variation over seasons (Tefera et al. 2022). However, integrating bird community and habitat types study can provide deeper insights into community assembly mechanisms and their implications for bird strike prevention (Jia et al. 2020). For instance, different habitat types support distinct bird communities: wetlands and forests tend to support higher species richness, whereas farmlands often attract large numbers of granivorous birds, potentially elevating collision risks (Santos et al. 2024; Weideman et al. 2020). Despite this, few studies have combined bird diversity and environment to analyze how ecological processes shape bird communities in airports or how habitat features influence these patterns.

Studies on airport bird communities are typically conducted at three spatial scales: fine‐scale, local, and regional. Wildlife management strategies are typically implemented separately at each airport, as wildlife hazards vary significantly between different locations (Conkling et al. 2018). Many studies have focused on fine‐scale investigations, examining species composition and strike risk at individual airports (Chen et al. 2023; Yuan et al. 2023). Such as the RobotFalcon system in Workum, Netherlands, which demonstrated immediate and sustained dispersal of bird flocks (Storms et al. 2022). Integrated analyses of bird strikes, weather radar, and eBird data across three New York airports establish that migration intensity and species occurrence data can effectively predict strike probability (Nilsson et al. 2021). The motion‐activated camera surveillance of Irish hares ( *Lepus timidus* ) at Dublin International Airport reveals significant temporal correlations between hare activity patterns and strike incidents (Ball et al. 2023).

At the regional scale, research has emphasized macroecological drivers such as climatic factors and elevation gradients (Pfeiffer et al. 2018; Steele and Weston 2021). Key findings include GPS tracking studies in Spain that identified spatiotemporal overlap between general aviation operations and peak activity periods of high‐risk bird species (Arrondo et al. 2021), as well as analyses of U.S. airport strike data demonstrating the significant influence of species‐specific traits on collision frequency (Fernandez‐Juricic et al. 2018). While many studies have investigated bird communities at individual airports (fine‐scale), comparative local‐scale investigations, examining multiple airports within a shared ecological region, remain rare, despite their potential to reveal how similar habitats shape bird communities and inform more effective, regionally adapted risk management strategies.

**FIGURE 1:**
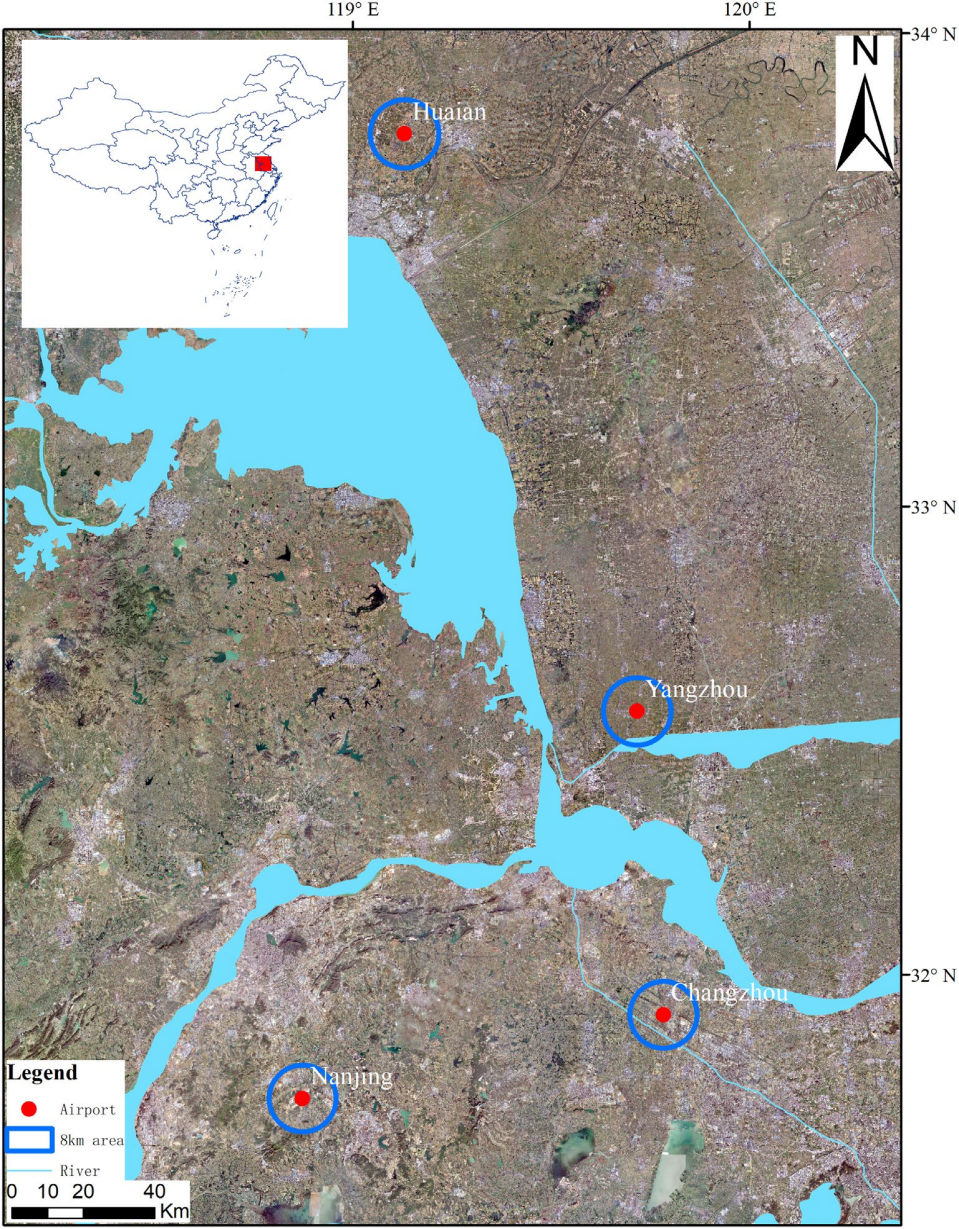
Locations of study sites where airports are shown as red dots. The top‐left map shows the location of the study area in eastern China. The red solid circles indicate the locations of the airports, while the blue hollow circles represent the 8 km surrounding radius around each airport. The names of the airports are labeled in white text.

This study addresses this gap by examining bird diversity and community assembly mechanisms at four airports in the Lower Yangtze River Plain (Figure 1). This region features heterogeneous landscapes, including farmland, woodlands, wetlands, and urban areas (Chen et al. 2023). These landscapes are undergoing rapid transformation due to urbanization, altering bird diversity and increasing the likelihood of bird‐aircraft collisions (Chen et al. 2025). For example, farmland provides food resources that attract high‐abundance species, while wetlands and woodlands support diverse but less abundant communities (Gao et al. 2021). Since bird strike risks are directly linked to both species diversity and abundance (Blackwell et al. 2019; Coccon et al. 2015).

In recent decades, rapid economic development and urbanization in the Yangtze River Delta region have led to a significant expansion of impermeable surfaces (Chen et al. 2025). Airports are densely distributed across the Lower Yangtze River Plain, and the surrounding areas have undergone rapid urbanization, resulting in substantial habitat changes and significant shifts in bird diversity patterns (Chen et al. 2024). While macroecological drivers, such as climatic factors and elevation gradients, have been well‐studied at broader scales (Pfeiffer et al. 2018; Steele and Weston 2021), research focusing on bird communities at the local scale, particularly within airport areas, remains scarce. By examining bird diversity in multiple airports and their surrounding habitats, we aim to uncover the similarities and differences in bird communities shaped by comparable ecological divergence. This study addresses three primary objectives: (1) to characterize the composition and structure of bird communities at airports in the Lower Yangtze River Plain; (2) to determine whether bird communities differ among airports within this shared biogeographical region; and (3) to identify the ecological and environmental factors driving any observed differences.

## 2 Materials and Methods

### 2.1 Study Area

This study focused on four airports (Changzhou Benniu International Airport, CZ; Huai'an Lianshui International Airports, HA; Nanjing Lukou International Airport, NJ; and Yangzhou Taizhou International Airport, YT) in Jiangsu Province, China, situated in the highly developed Yangtze River Delta plain within the lower reaches of the Yangtze River (Chen et al. 2024). The study area is located along the East Asian–Australasian Flyway (EAAF), making it internationally significant for migratory bird conservation. Geographically, the region lies at the confluence of the Yangtze River, Huai River, and Yellow Sea, encompassing major water bodies including Tai Lake and Hongze Lake, which are China's third and fourth largest freshwater lakes (Chen et al. 2025). Climatically, the area represents a transitional zone between subtropical and warm‐temperate climates, characterized by distinct seasonal variations and pronounced monsoon influences (Chen et al. 2024). Meteorological records indicate mean annual temperatures ranging from 13.6°C to 16.1°C with annual precipitation between 704 and 1250 mm. These climatic conditions, combined with diverse aquatic and terrestrial ecosystems, support rich biodiversity and provide suitable habitats for numerous wildlife species.

Habitats data around the airports were obtained from the National Earth System Science Data Center for the Yangtze River Delta region through the national science and technology resource sharing service platform (http://nnu.geodata.cn:8008), with a resolution of 30 × 30 m per grid unit. QGIS 3.16.9 was used for image interpretation of the layers (QGIS Development Team 2021). To better characterize bird habitat requirements, the original land cover types were reclassified into four predominant habitat categories: (1) farmland, (2) forestland, (3) wetland, and (4) residential area. These four matrix types account for distinct ecological functions and resource availability critical for different bird guilds, while maintaining representation of dominant landscape matrices commonly used in bird studies (Chen et al. 2025).

### 2.2 Bird Surveys

**FIGURE 2:**
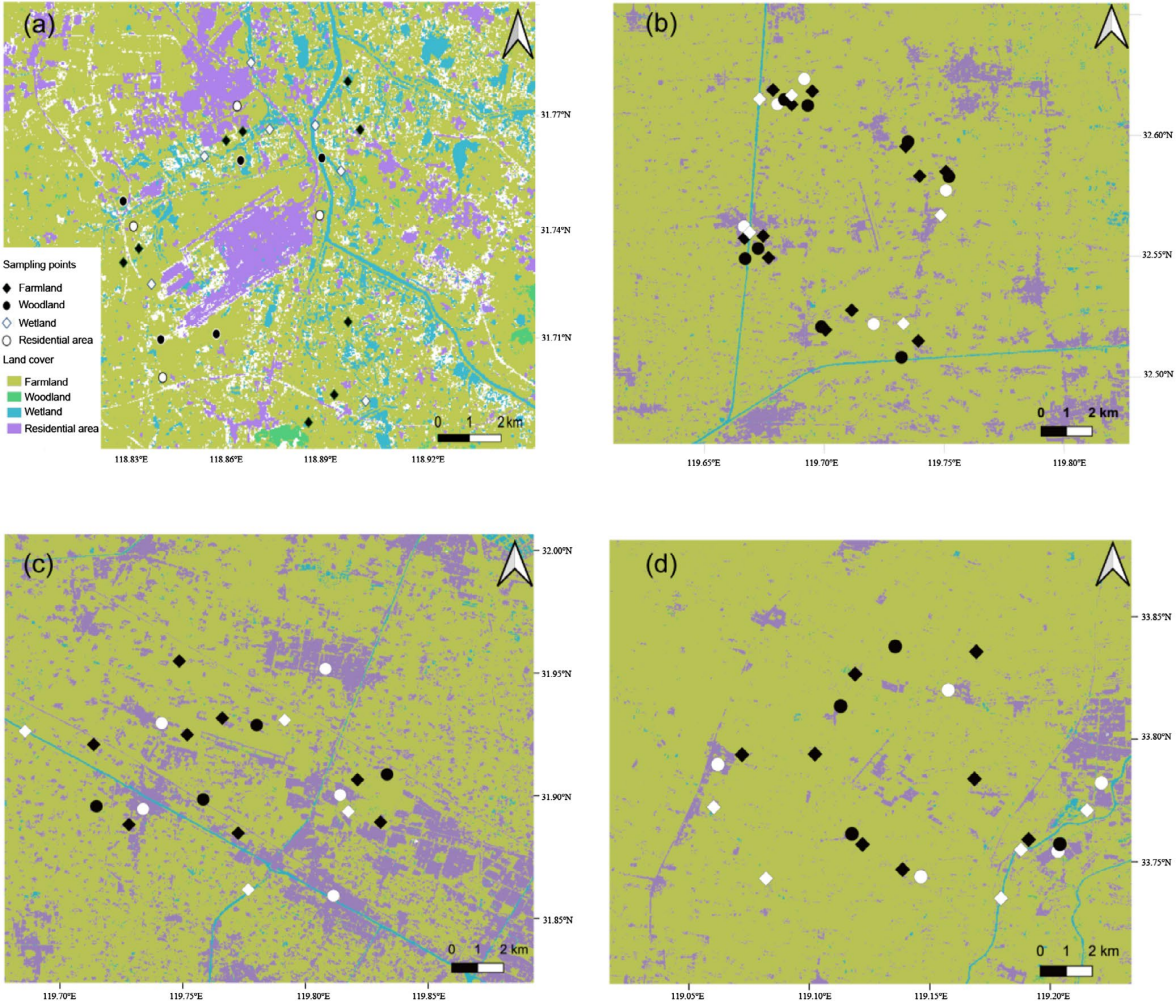
Location of the sampling points and landscape types at the four airports. (a) Nanjing Lukou International Airport; (b) Yangzhou Taizhou International Airport; (c) Changzhou Benniu International Airport; (d) Huai'an Lianshui International Airports.

From November 2018 to October 2019, we conducted monthly bird point‐count surveys across all four study airports using a standardized protocol to assess species richness and abundance patterns (Buckland 2006). Using a stratified random sampling design, we systematically surveyed four dominant habitat types at each airport (farmland, woodland, wetland, and residential area), with survey points geographically distributed to ensure representative habitat coverage (Figure 2). A total of 25, 30, 21, and 22 bird survey sampling points were used at NJ, YT, CZ, and HA, respectively (Table 1; Figure 2).

**TABLE 1:** Proportion of four habitat types within 8 km of four Yangtze River Delta airports and the number of bird sampling points per each habitat type.

Airport	Percentage of total area occupied by each habitat type				Numbers of sampling points in habitats				Total number
	Farmland (%)	Wetland (%)	Woodland (%)	Residential area (%)	Farmland	Wetland	Woodland	Residential area	
NJ	69.66	8.42	15.05%	6.87	9	7	5	4	25
YT	88.01	1.13	0.07	10.79	12	5	8	5	30
CZ	74.29	1.61	0.39	23.71	8	4	4	5	21
HA	91.56	0.67	0.25	0.52	8	5	4	5	22

Abbreviations: CZ, Changzhou Benniu International Airport; HA, Huai'an Lianshui International Airport; NJ, Nanjing Lukou International Airport; YT, Yangzhou Taizhou International Airport.

All surveys were performed by trained dual observers during optimal detection conditions: on fair weather days (no precipitation, fog, or winds > 15 km/h) within peak activity periods (30 min post‐dawn to 10:00 and 15:30 to 30 min pre‐sunset). Each 20‐min stationary point count encompassed 360° observations within fixed 30 m radius plots using 8 × 42 binoculars, with all visual and auditory detections recorded while minimizing observer disturbance. Species identification was verified using regional field guides (Zheng 2017), and we documented precise location data (GPS coordinates), habitat types, species names, and number of individuals for each plot.

The study area is influenced by subtropical monsoons and has distinct four seasons; the data collection and classification for the research are carried out accordingly. Specifically, the data from March to May are classified as the spring survey data, those from June to August are categorized as the summer survey data, the data from September to November are regarded as the autumn survey data, and the data from December to February of the following year are considered as the winter survey data.

### 2.3 Statistical Analysis

To validate sampling adequacy, we generated sample coverage curves using the “iNEXT” package (Hsieh et al. 2016), and constructed species accumulation curves (SAC) through 1000 permutations in the vegan package (Ugland et al. 2003). These analyses confirmed our survey effort sufficiently captured bird community composition, with SACs reaching asymptotic plateaus for each airport.

We calculated species richness, Shannon–Wiener index, Simpson's diversity index, Bray–Curtis similarity, and the sampling adequacy analysis using the “vegan” package (Oksanen et al. 2019). All data were summarized per plot per habitat type during each season. Due to the non‐normal distribution of our data, we employed Kruskal–Wallis rank sum tests followed by Bonferroni‐adjusted post hoc multiple comparisons to examine differences between each airport in mean species richness, abundance, Shannon–Wiener index, and Simpson's diversity index across habitat types within each plot. We used PERMANOVA (permutational multivariate analysis of variance) with 999 permutations to test the joint effects of Airport, habitat, and season on species richness patterns. Resemblance matrices were based on zero‐inflated Bray–Curtis similarity measures. Differences were tested using Analysis of Similarities (ANOSIM), and where differences existed, these were explored using the test of homogeneity in Permutational Dispersions (PERMDISP). The significance level was set at *α* = 0.05. All analyses were performed in R 4.0.1 (R Core Team 2020).

## 3 Results

**FIGURE 3:**
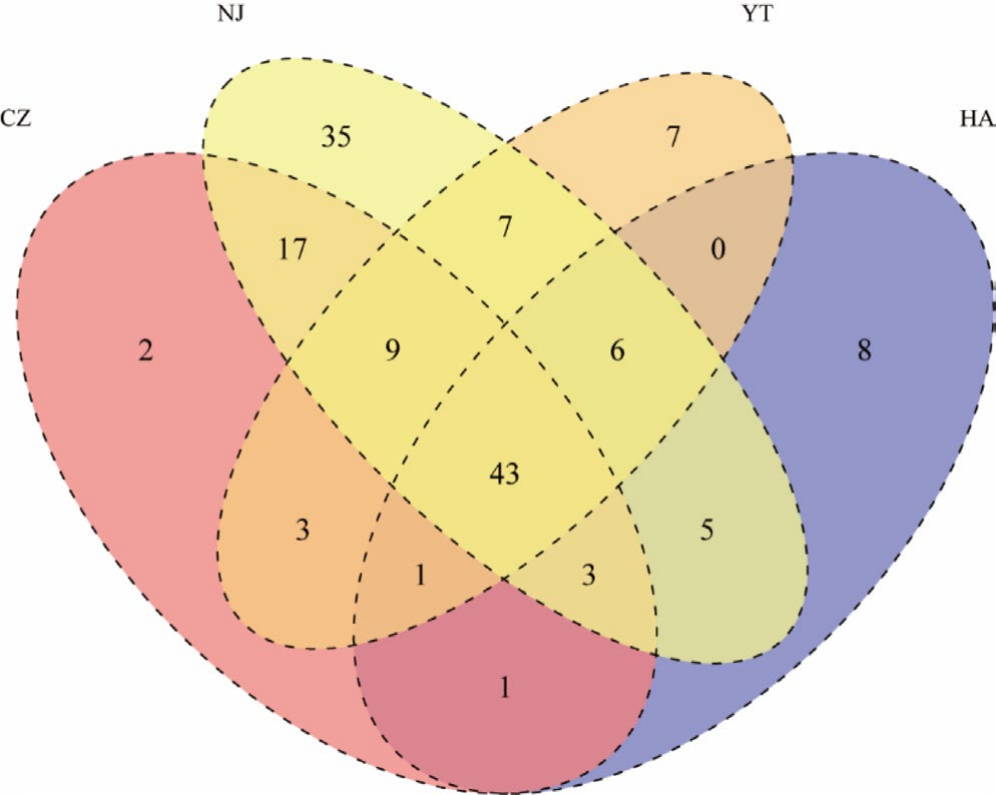
Venn diagram representing the number of bird species and the number of shared species at the four airports. CZ, Changzhou Benniu International Airport; HA, Huai'an Lianshui International Airport; NJ, Nanjing Lukou International Airport; YT, Yangzhou Taizhou International Airport.

The SAC leveled off, indicating that the survey had captured most of the bird species present (Figure S1). The survey across the four airports discovered a total of 147 bird species. Specifically, NJ had 125 species, CZ had 79 species, YT had 76 species, and HA had 67 species, with a total of 43 shared species among them. NJ had the highest number of unique species, with 35, while CZ, HA, and YT had lower numbers of unique species, with 2, 8, and 7 species respectively (Figure 3). Among these species, the Rustic Bunting ( *Emberiza rustica* ) is listed as Vulnerable (VU) on the IUCN Red List of Threatened Species, while the Northern Lapwing ( *Vanellus vanellus* ), Reed Parrotbill ( *Paradoxornis heudei* ), and Japanese Quail ( *Coturnix japonica* ) are categorized as Near Threatened (NT). The remaining species are classified as Least Concern (LC) (see Appendix S2). Of the total species recorded, 50 were permanent residents while 96 were migrants.

**FIGURE 4:**
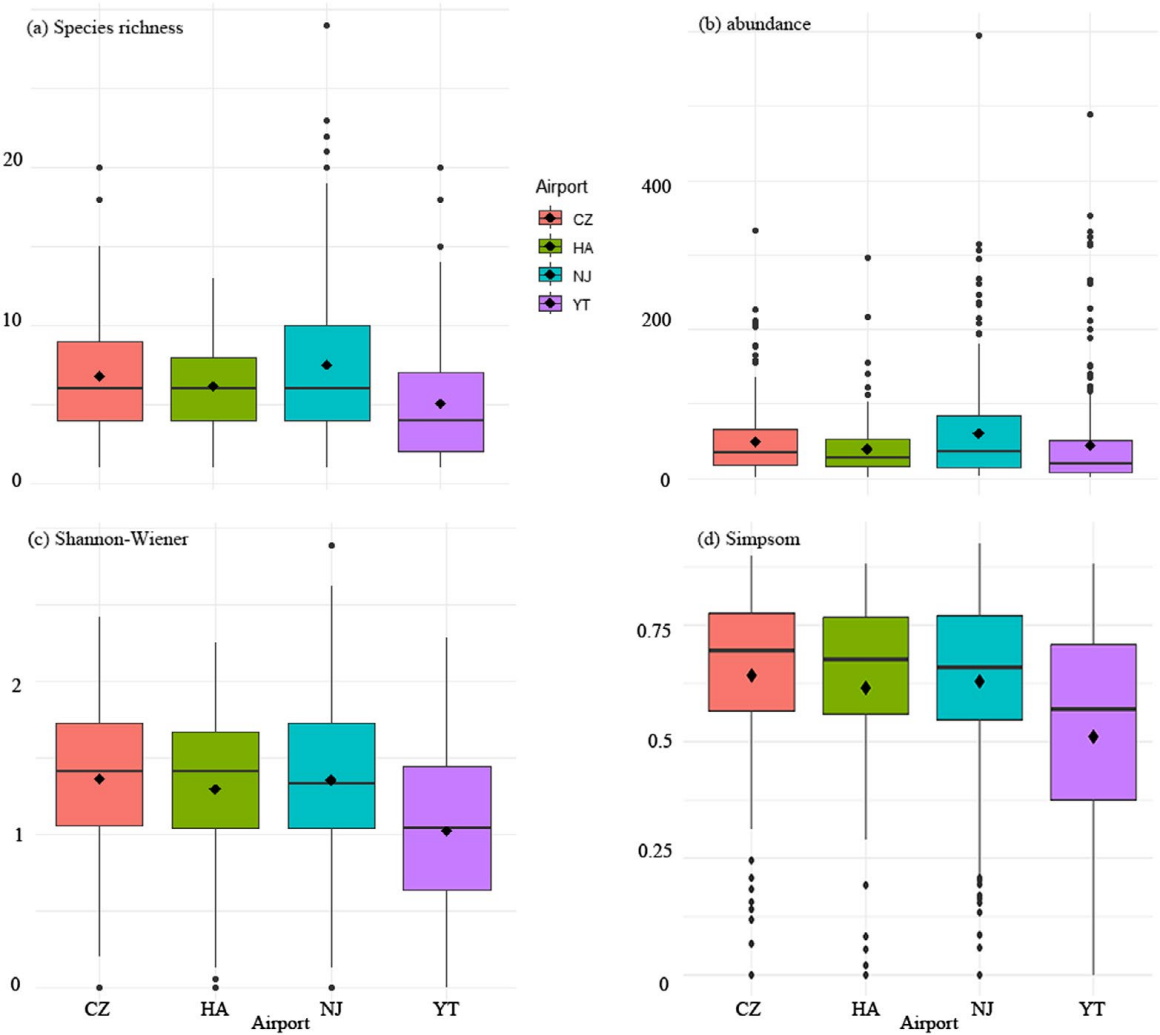
Differences in bird community among four Yangtze River Delta airports. (a) species richness; (b) number of individuals; (c) Shannon–Wiener index (*H*′); (d) Simpson's diversity index. CZ, Changzhou Benniu International Airport; HA, Huai'an Lianshui International Airport; NJ, Nanjing Lukou International Airport; YT, Yangzhou Taizhou International Airport.

The four airports exhibit significant differences in habitat composition and landscape characteristics within an 8 km radius, particularly in the proportions of farmland, wetland, and residential areas (Table 1). Bird community diversity varied notably across the four airports, suggesting distinct environmental characteristics or habitat qualities at each site. Specifically, bird species richness (*H* = 61.25, df = 3, *p* < 0.01; NJ^a^ CZ^a^ YT^b^ HA^b^), abundance (*H* = 30.86, df = 3, *p* < 0.01; NJ^a^ CZ^a^ YT^ab^ HA^ab^), Shannon–Wiener index (*H* = 50.49, df = 3, *p* < 0.01; CZ^a^ NJ^a^ YT^b^ HA^c^), and Simpson's diversity index (*H* = 42.25, df = 3, *p* < 0.01; CZ^a^ NJ^a^ YT^b^ HA^c^) differed significantly among the four airports (Figure 4). Post hoc comparisons indicated that NJ generally exhibited the highest species richness and abundance, while CZ and HA varied significantly in diversity indices.

Habitat type and season also played important roles in shaping bird community structure. Across habitats and seasons, bird diversity metrics showed significant variation (Kruskal–Wallis tests, all *p* < 0.01), implying that local habitat features and seasonal dynamics influence bird presence and activity levels. From a seasonal perspective, both species richness and diversity were higher in spring and summer compared to autumn and winter (Figure S2). In terms of habitat types, woodlands supported greater bird species richness and diversity than farmlands, wetlands, and residential areas (Figure S3). This pattern may be attributed to the small size and fragmented nature of wetlands surrounding the airport, which likely reduced their capacity to sustain diverse bird communities (Figure 2).

The combined effects of airport, habitat, and season accounted for a substantial proportion of variation in bird community composition. PERMANOVA revealed significant overall effects of these factors and their interactions on species richness (*F* = 3.57, *R* ^2^ = 0.23, *p* < 0.001, 999 permutations), abundance (*F* = 2.51, *R* ^2^ = 0.18, *p* = 0.002), Shannon–Wiener index (*F* = 3.81, *R* ^2^ = 0.24, *p* < 0.001), and Simpson's diversity index (*F* = 3.53, *R* ^2^ = 0.23, *p* < 0.001), and showed that 23%, 18%, 24%, and 23% of the variation in bird community composition was explained by these factors and their interactions. These results highlight the multifactorial nature of bird community structuring in airport environments.

**FIGURE 5:**
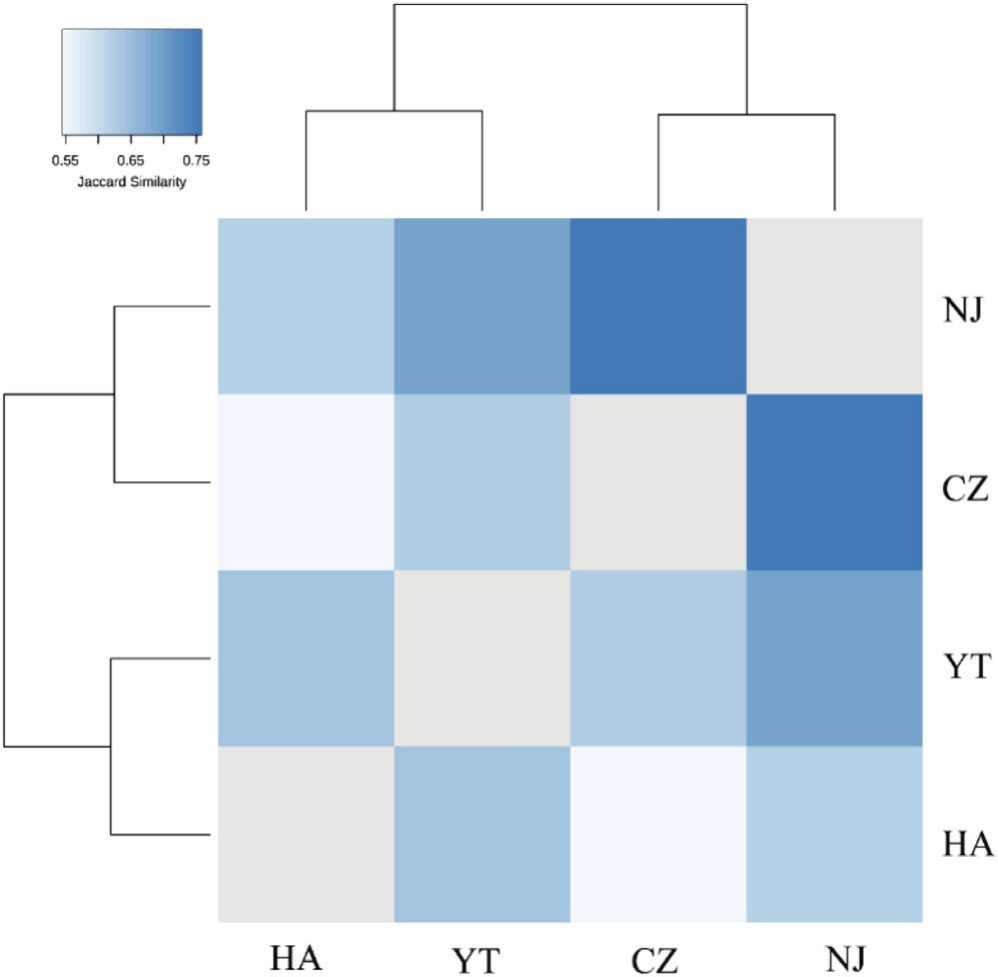
Distance tree based on the Jaccard community similarity index. CZ, Changzhou Benniu International Airport; HA, Huai'an Lianshui International Airport; NJ, Nanjing Lukou International Airport; YT, Yangzhou Taizhou International Airport.

Bird community composition was not only quantitatively but also qualitatively distinct among airports. The assemblage of bird community composition differed significantly among the four airports (ANOSIM: Global *R* = 0.051, *p* = 0.001). Pairwise comparisons revealed significant differences in three of the six airport pairs: CZ versus HA (*R* = 0.125, *p* = 0.001), HA versus NJ (*R* = 0.138, *p* = 0.001), and NJ versus YZ (*R* = 0.076, *p* = 0.001), while the remaining pairs showed no significant assemblage differences. Furthermore, PERMDISP revealed significant differences in beta diversity dispersion among the four airports (*F*_3,808_ = 13.119, *p* < 0.001), particularly with CZ airport differing significantly from all others (all *p* < 0.05), and YT airport differing from both HA (*p* = 0.017) and NJ (*p* = 0.001). Spatial similarity patterns further supported the distinctiveness of bird communities across airports. Bray–Curtis similarity was highest between NJ and HA (BC = 0.67), suggesting comparable community structures, whereas YT and CZ showed the least similarity (BC = 0.27), indicating divergent ecological conditions (Table 2). Species‐sharing data and a Jaccard‐based distance tree also illustrated these trends, with NJ and CZ sharing the greatest number of common species (72 species), reinforcing their relative ecological resemblance (Table 2; Figure 5).

**TABLE 2:** Bray–Curtis similarity index (lower left) and the number of common species (upper right).

	CZ	HA	NJ	YT
CZ		48	72	56
HA	0.50		57	50
NJ	0.40	0.67		65
YT	0.27	0.48	0.36	

Abbreviations: CZ, Changzhou Benniu International Airport; HA, Huai'an Lianshui International Airport; NJ, Nanjing Lukou International Airport; YT, Yangzhou Taizhou International Airport.

## 4 Discussion

This study demonstrates that bird communities at airports within the same biogeographic region can exhibit marked differences in species richness, abundance, and composition. These variations reflect differences in habitat heterogeneity, landscape context, and anthropogenic disturbance surrounding each airport. For example, the high species richness and abundance observed at NJ airport may be attributed to its relatively diverse and well‐preserved surrounding habitats, which support both migratory and resident bird species (Table 1, Figure 1). In contrast, airports like HA and YT, with more urbanized or homogeneous landscapes, may offer fewer foraging and nesting opportunities, resulting in lower diversity. These findings underscore the importance of considering local environmental features when assessing bird strike risks. While previous studies have often focused on single‐airport assessments or macroecological factors, our multi‐airport comparison at a local scale reveals how ecological divergence at the landscape level can shape distinct bird assemblages, even within a shared climatic zone. This has important implications for airport‐specific bird management strategies, which must be adapted to the unique ecological context of each site.

### 4.1 Global Comparisons of Bird Diversity at Airports

The bird species and community patterns observed in this study reflect broader trends observed at airports worldwide, although regional variations highlight the influence of local ecological factors on bird diversity. Our findings are consistent with those of studies conducted at airports near wetland habitats in Europe that reported higher bird diversity at European airports with nearby wetlands (Iglay et al. 2017). Similarly, at NJ, the presence of adjacent wetlands supports a diverse bird community, in line with these broader regional trends. In North America, airports situated near agricultural lands, particularly those with substantial populations of granivorous birds, also exhibit increased bird abundance, correlating directly with heightened strike risks (Conkling et al. 2018). For example, U.S. airports located in agricultural regions of the Midwest report high frequencies of bird species such as Canada Geese and American Robins, which are known contributors to bird strike incidents (Dolbeer et al. 2014). This pattern mirrors our observations at airports near agricultural zones in the Yangtze Delta, where certain bird species are frequently implicated in strikes.

Contrasting with the higher bird diversity seen at European and North American airports, Australian airports, particularly those in coastal regions, tend to report lower overall bird diversity (Alquezar et al. 2020). However, these airports face significant strike risks due to large populations of waterfowl and migratory shorebirds (Alquezar et al. 2020). This highlights the importance of habitat‐specific dynamics and the fact that lower bird diversity does not necessarily equate to lower strike risks, as the composition of bird species also plays a crucial role in collision rates. In South Africa, research at Oliver Tambo International Airport underscores the importance of effective land‐use planning within airport buffer zones to mitigate bird strike risks (Robinson et al. 2021). These findings align with our study's recommendation for more integrated planning in areas with higher bird activity, especially near wetland and agricultural zones. In Ethiopia, the study of Bahir Dar International Airport recorded 80 bird species (15 orders, 40 families), with modified habitats showing peak diversity (*H*′ = 3.59) and evenness (*E* = 0.96) during wet seasons (Tefera et al. 2022). Comparative analysis reveals Bahir Dar International Airport's bird species richness (80 species) falls between NJ (125 species) and the other three Yangtze Delta airports (67–79 species) in our study. Notably, Bahir Dar's modified habitats achieved higher wet‐season diversity metrics (*H*′ = 3.59, *E* = 0.96) than any single habitat type in our study area, suggesting human‐altered landscapes may unexpectedly support robust bird communities in tropical climates despite lower overall species counts. Both studies consistently identify temporal (seasonal/wet‐dry) and spatial (habitat‐type) factors as critical drivers of airport bird distribution patterns.

### 4.2 Inter‐Airport Variation Within the Lower Yangtze River Plain

Despite the four airports being located within the same bioregion—the Lower Yangtze River Plain, significant differences in bird community composition were observed (Figure 4). This variation highlights the importance of local ecological factors, such as habitat type and land use practices, in shaping bird communities (García‐Navas and Thuiller 2020). Species richness is often influenced by habitat type (Magle et al. 2012), and this study confirms that different habitat types at the four airports significantly shape the bird communities present. Generally, residential areas, characterized by intense human activity, tend to have lower species richness compared to habitats like farmland, forests, and wetlands, which offer more suitable conditions for various bird species (McKinney 2006; Santos et al. 2024). At YT, CZ, and HA, the number of bird species in residential areas was lower than in other habitats (Figure S3), demonstrating the impact of urbanization on local bird diversity. This is consistent with findings from residential areas worldwide, where anthropogenic factors, such as habitat destruction and noise pollution, reduce species diversity (McKinney 2006). However, the bird species count in the residential area of NJ was higher than in the other three habitat types, which can be attributed to the unique habitat distribution around the airport. Lukou Airport is surrounded by a diverse mix of habitats, including farmland, forests, and wetlands, which support generalist species and provide multiple resources, such as food and nesting sites, enhancing species richness in residential areas (Table 1). In contrast, HA, situated in a more rural, monocultural landscape, exhibited the lowest number of bird species among the four airports. This pattern of variation underscores the complex relationship between landscape features and bird behavior, even within the same biogeographical region. Moreover, airports like NJ and CZ, despite being geographically close, showed marked differences in their bird communities. This suggests that fine‐scale habitat features, such as the availability of foraging resources, nesting sites, and habitat connectivity, can significantly influence local bird populations (Santos et al. 2024).

### 4.3 Effects of Land Use and Habitat Type on Bird Communities

Land use composition, particularly the proportion and configuration of farmland, woodland, wetland, and residential areas, had pronounced effects on bird community structure. Woodland habitats supported the highest bird diversity, consistent with their structural complexity and resource availability (Figure S3). These patterns were most prominent at NJ and CZ, where diverse habitat mosaics likely promoted higher habitat connectivity and foraging options. Woodlands were found to support a higher diversity of bird species compared to farmlands, which were dominated by fewer species but in larger numbers (Sayol et al. 2021). This finding aligns with previous research showing that habitats rich in plant diversity, such as wetlands and woodlands, provide more foraging and nesting opportunities, thus supporting greater species diversity (Basile et al. 2021). Wetlands, in particular, offer an abundance of resources, including water, food, and shelter, which attract a diverse array of bird species. These habitats support both resident and migratory birds, contributing to higher overall species richness (Cheng et al. 2022; Ma et al. 2023). In contrast, agricultural habitats, while providing abundant food resources, tend to support larger flocks of granivorous species, which can result in increased collision risks, especially during peak migration periods (Anderle et al. 2023; Liao et al. 2024). Species such as the Eurasian Tree Sparrow ( *Passer montanus* ) and Common Starling ( *Sturnus vulgaris* ), which are often abundant in agricultural landscapes, are frequently involved in bird strikes at airports (Chen et al. 2023; Yuan et al. 2023).

### 4.4 Seasonal Dynamics and Implications for Bird Strike Risk

Seasonality emerged as a critical factor influencing bird diversity and abundance across the four airports. Species richness and diversity were consistently higher in spring and summer (Figure S2). This study area lies within the East Asian–Australasian Flyway (EAAF), a critical migratory route for numerous bird species. Of the total species recorded in this study, 50 were permanent residents, while 96 were migratory species. This highlights the significant role of migratory birds in the bird communities at these airports. Our results indicate that bird communities in the studied airports are highly dynamic, with notable seasonal variations in species composition and abundance (Figure S2). This seasonal fluctuation in bird community structure is consistent with findings from other regions (Leveau et al. 2015). The presence of large numbers of migratory birds during specific times of the year increases the likelihood of bird strikes, necessitating proactive measures during peak migration seasons (Steele and Weston 2021; Waugh et al. 2011). Given the seasonal fluctuations in bird activity, particularly during migratory periods, airport bird strike prevention strategies must account for these changes to effectively reduce risks (Dolbeer and Wright 2009; Vaishnav et al. 2024). Future research should continue to explore how fine‐scale habitat features, such as vegetation type and land use, influence both bird diversity and strike risks, ultimately guiding more effective mitigation measures for bird strikes at airports.

## 5 Conclusions

This study provides a multi‐airport, local‐scale analysis of bird communities in the Lower Yangtze River Plain, revealing how spatial and temporal habitat heterogeneity drives significant variation in species richness, abundance, and composition—even within the same biogeographic zone. By combining species‐level data with environmental attributes, we demonstrate that bird community cannot be generalized across airports; rather, it is shaped by localized factors such as land‐use composition, seasonal dynamics, and habitat configuration. These findings emphasize the importance of site‐specific risk assessments that move beyond species counts to consider ecological context. A key contribution of this study is the identification of woodland and wetland habitats as biodiversity hotspots within airport environments, supporting higher bird diversity during migration periods. Furthermore, the unexpectedly high richness in certain residential zones, especially at NJ airport, suggests that fine‐scale habitat features, including land use and habitat type, deserve greater attention in future risk modeling and mitigation strategies. This study used 30‐m resolution satellite data may overlook small‐scale habitat features within residential areas (e.g., backyard vegetation, and street trees), which could affect bird species richness and communities. Future research should expand the geographic scope of studies while incorporating higher‐resolution imagery or ground‐based surveys to better capture fine‐grained urban heterogeneity. Integrating ecological insights into airport safety protocols will be critical for mitigating bird strike risks while protecting biodiversity.

## Author Contributions

**Wan Chen:** formal analysis (equal), software (equal), writing – original draft (equal). **Caiqian Sun:** conceptualization (equal), data curation (equal), funding acquisition (equal), investigation (equal). **Yuanyuan Cai:** data curation (equal), software (equal). **Hang Zhang:** data curation (equal), formal analysis (equal). **Weiya Qian:** data curation (equal), formal analysis (equal). **Peng Li:** investigation (equal), methodology (equal). **Xinglong Huang:** data curation (equal), formal analysis (equal). **Qing Chang:** conceptualization (equal), data curation (equal), supervision (equal). **Chaochao Hu:** conceptualization (equal), data curation (equal), formal analysis (equal), funding acquisition (equal), writing – original draft (equal).

## Conflicts of Interest

The authors declare no conflicts of interest.

## Supporting information


Appendix S1:

Appendix S2:

## Data Availability

All relevant data have been provided in the Appendix S2 (Appendix.csv) uploaded with the submission and has been designated as Supporting Information for review and publication.
