# Supplementary material for: Divergent Bird Diversity Patterns Among Four Airports in the Same Bioregion: Assessing Local‐Scale Drivers of Bird Community Assembly
Source: Ecol Evol. 2025 Jul 13;15(7):e71772. doi: 10.1002/ece3.71772 (PMC12256201; doi:10.1002/ece3.71772)
Supplement: Supplementary file 1 — Appendix S1: [file ECE3-15-e71772-s002.docx]

## Figure S1

Sampling coverage curves of 4 airports. “interpolated” indicates the curve of bird survey (solid line) and “extrapolated” indicates sample coverages are up to the coverage value of double the reference sample size (dashed line).


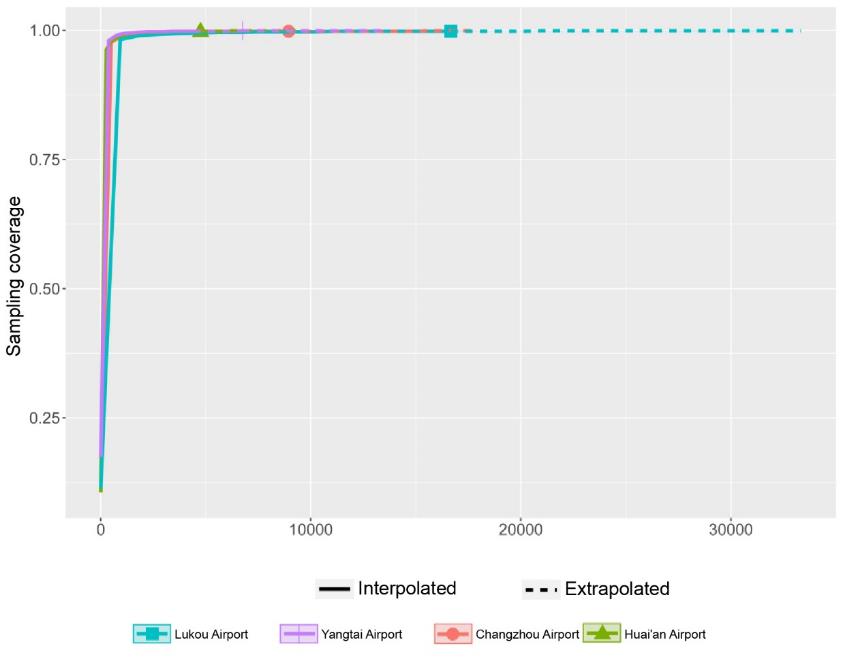


## Figure S2

Seasonal variations in bird community among four Yangtze River Delta airports. (a) species richness; (b) number of individuals; (c) Shannon-Wiener index; (d) Simpson’s diversity index.

(b)

(a)


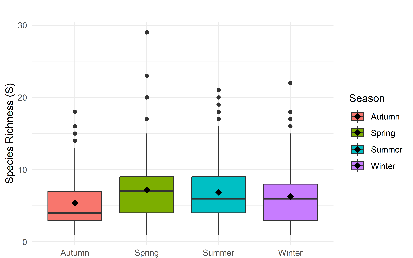

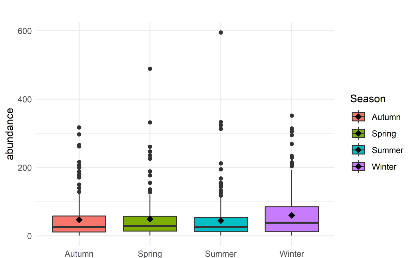


(d)

(c)


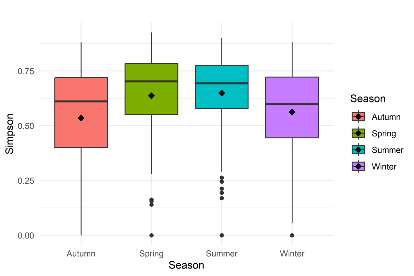

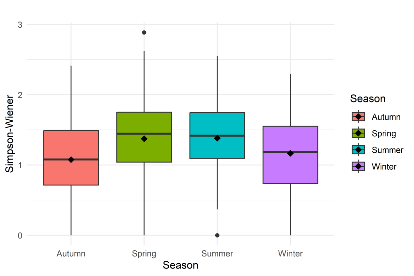


## Figure S3

Differences in bird communities among four habitat types at Yangtze River Delta airports. (a) species richness; (b) number of individuals; (c) Shannon-Wiener index; (d) Simpson’s diversity index.

(b)

(a)


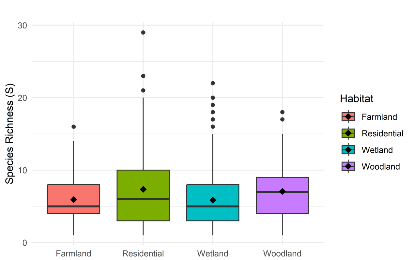

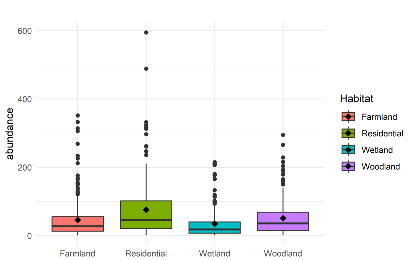


(d)

(c)


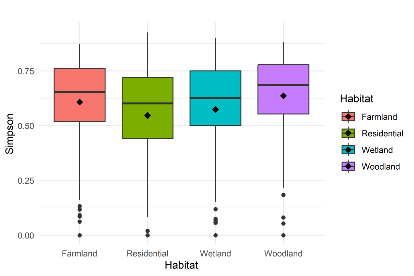

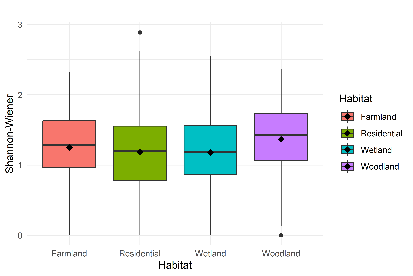


## Appendix 1

A checklist on birds and number of individuals each species at four Airport.

| English name | Scientific name | IUCN | CZ | HA | NJ | YT |
| --- | --- | --- | --- | --- | --- | --- |
| Northern Goshawk | *Accipiter gentilis* | LC |  |  | 1 |  |
| Crested Myna | *Acridotheres cristatellus* | LC | 971 | 59 | 1786 | 744 |
| Oriental Reed Warbler | *Acrocephalus orientalis* | LC | 4 |  | 2 |  |
| Common Sandpiper | *Actitis hypoleucos* | LC |  |  | 4 | 3 |
| Black-throated Bushtit | *Aegithalos concinnus* | LC | 1 |  |  |  |
| Silver-throated Bushtit | *Aegithalos glaucogularis* | LC | 15 |  | 117 | 39 |
| Eurasian Skylark | *Alauda arvensis* | LC | 11 | 17 | 320 | 199 |
| Oriental Skylark | *Alauda gulgula* | LC |  |  | 113 |  |
| Common Kingfisher | *Alcedo atthis* | LC | 5 | 2 | 5 | 1 |
| White-breasted Waterhen | *Amaurornis phoenicurus* | LC | 2 | 1 |  | 10 |
| Eurasian Teal | *Anas crecca* | LC |  |  | 48 |  |
| Mallard | *Anas platyrhynchos* | LC |  |  | 13 |  |
| Chinese Spot-billed Duck | *Anas zonorhyncha* | LC |  | 18 | 36 |  |
| Olive-backed Pipit | *Anthus hodgsoni* | LC | 24 | 9 | 292 | 39 |
| Richard's Pipit | *Anthus richardi* | LC |  |  | 12 | 2 |
| Buff-bellied Pipit | *Anthus rubescens* | LC | 1 |  |  |  |
| Great Egret | *Ardea alba* | LC |  |  | 6 |  |
| Grey Heron | *Ardea cinerea* | LC | 1 |  | 4 |  |
| Intermediate Egret | *Ardea intermedia* | LC | 9 | 5 | 6 |  |
| Purple Heron | *Ardea purpurea* | LC |  | 1 |  |  |
| Chinese Pond Heron | *Ardeola bacchus* | LC | 184 | 48 | 97 | 138 |
| Little Owl | *Athene noctua* | LC |  | 3 |  |  |
| Chinese Bamboo Partridge | *Bambusicola thoracicus* | LC |  |  | 6 |  |
| Cattle Egret | *Bubulcus coromandus* | LC | 498 | 122 | 383 | 73 |
| Green-backed Heron | *Butorides striata* | LC |  | 1 | 1 | 2 |
| Long-toed Stint | *Calidris subminuta* | LC |  |  | 2 |  |
| Grey Nightjar | *Caprimulgus jotaka* | LC |  |  | 1 |  |
| Red-rumped Swallow | *Cecropis daurica* | LC | 80 | 189 | 91 | 61 |
| Lesser Coucal | *Centropus bengalensis* | LC |  |  | 1 |  |
| Pied Kingfisher | *Ceryle rudis* | LC | 3 |  | 13 |  |
| Kentish Plover | *Charadrius alexandrinus* | LC | 7 |  | 7 |  |
| Greater Sand Plover | *Charadrius leschenaultii* | LC | 16 |  | 2 |  |
| Long-billed Plover | *Charadrius placidus* | LC |  |  | 8 |  |
| Oriental Plover | *Charadrius veredus* | LC |  |  |  | 5 |
| Whiskered Tern | *Chlidonias hybrida* | LC |  |  | 1 |  |
| Oriental Greenfinch | *Chloris sinica* | LC | 50 | 2 | 92 | 4 |
| Black-headed Gull | *Chroicocephalus ridibundus* | LC |  |  |  | 1 |
| Zitting Cisticola | *Cisticola juncidis* | LC |  |  | 3 |  |
| Rock Dove | *Columba livia* | LC | 98 | 792 | 126 | 625 |
| Oriental Magpie-Robin | *Copsychus saularis* | LC |  |  |  | 1 |
| Japanese Quail | *Coturnix japonica* | NT |  |  |  | 2 |
| Common Cuckoo | *Cuculus canorus* | LC | 3 | 2 | 1 | 3 |
| Indian Cuckoo | *Cuculus micropterus* | LC |  | 5 | 3 | 5 |
| Lesser Cuckoo | *Cuculus poliocephalus* | LC |  |  | 1 |  |
| Himalayan Cuckoo | *Cuculus saturatus* | LC |  |  | 1 |  |
| Azure-winged Magpie | *Cyanopica cyanus* | LC | 35 | 217 | 20 | 130 |
| Great Spotted Woodpecker | *Dendrocopos major* | LC |  | 2 |  |  |
| Black Drongo | *Dicrurus macrocercus* | LC | 18 | 16 | 73 | 40 |
| Little Egret | *Egretta garzetta* | LC | 164 | 48 | 706 | 123 |
| Black-shouldered Kite | *Elanus caeruleus* | LC |  | 1 | 1 |  |
| Yellow-browed Bunting | *Emberiza chrysophrys* | LC | 6 | 4 | 2 | 11 |
| Yellow-throated Bunting | *Emberiza elegans* | LC | 7 |  | 10 | 12 |
| Chestnut-eared Bunting | *Emberiza fucata* | LC |  |  | 3 | 7 |
| Pallas's Reed Bunting | *Emberiza pallasi* | LC |  |  | 1 |  |
| Little Bunting | *Emberiza pusilla* | LC | 1 |  | 18 |  |
| Rustic Bunting | *Emberiza rustica* | VU | 16 | 4 | 1 | 193 |
| Common Reed Bunting | *Emberiza schoeniclus* | LC |  |  | 1 |  |
| Black-faced Bunting | *Emberiza spodocephala* | LC | 74 | 1 | 184 | 118 |
| Tristram's Bunting | *Emberiza tristrami* | LC | 1 |  |  | 3 |
| Chinese Grosbeak | *Eophona migratoria* | LC | 76 | 34 | 82 | 228 |
| Japanese Grosbeak | *Eophona personata* | LC | 3 |  | 1 | 2 |
| Western Koel | *Eudynamys scolopaceus* | LC | 3 |  |  | 1 |
| Eastern Red-footed Falcon | *Falco amurensis* | LC |  |  | 61 |  |
| Merlin | *Falco columbarius* | LC |  |  | 4 |  |
| Peregrine Falcon | *Falco peregrinus* | LC | 1 |  | 1 |  |
| Hobby | *Falco subbuteo* | LC |  |  | 1 |  |
| Common Kestrel | *Falco tinnunculus* | LC |  | 2 | 7 | 2 |
| Yellow-rumped Flycatcher | *Ficedula zanthopygia* | LC |  |  | 1 |  |
| Brambling | *Fringilla montifringilla* | LC | 1 |  | 33 | 4 |
| Common Coot | *Fulica atra* | LC | 1 |  | 4 |  |
| Common Snipe | *Gallinago gallinago* | LC | 3 | 2 | 3 | 2 |
| Swinhoe’s Snipe | *Gallinago megala* | LC |  |  | 1 |  |
| Pintail Snipe | *Gallinago stenura* | LC |  |  | 7 | 16 |
| Common Moorhen | *Gallinula chloropus* | LC | 83 | 70 | 283 | 122 |
| Oriental Pratincole | *Glareola maldivarum* | LC |  |  | 5 |  |
| Black-collared Starling | *Gracupica nigricollis* | LC | 4 | 2 | 26 | 1 |
| White-throated Kingfisher | *Halcyon smyrnensis* | LC |  |  |  | 1 |
| Large Hawk-cuckoo | *Hierococcyx sparverioides* | LC |  | 1 |  |  |
| Common Hawk-cuckoo | *Hierococcyx varius* | LC |  |  | 1 |  |
| Barn Swallow | *Hirundo rustica* | LC | 252 | 271 | 397 | 262 |
| Manchurian Bush Warbler | *Horornis canturians* | LC |  | 3 |  |  |
| Cinnamon Bittern | *Ixobrychus cinnamomeus* | LC |  | 1 | 1 |  |
| Black Bittern | *Ixobrychus flavicollis* | LC |  |  | 2 |  |
| Yellow Bittern | *Ixobrychus sinensis* | LC |  | 1 | 1 | 5 |
| Brown Shrike | *Lanius cristatus* | LC | 12 | 19 | 55 | 9 |
| Long-tailed Shrike | *Lanius schach* | LC | 172 | 73 | 288 | 116 |
| Chinese Grey Shrike | *Lanius sphenocercus* | LC |  | 1 |  |  |
| Tiger Shrike | *Lanius tigrinus* | LC |  | 2 |  |  |
| White-rumped Munia | *Lonchura striata* | LC | 64 |  | 57 |  |
| Black Kite | *Milvus migrans* | LC |  |  | 4 |  |
| White Wagtail | *Motacilla alba* | LC | 36 | 43 | 219 | 57 |
| Grey Wagtail | *Motacilla cinerea* | LC | 1 |  | 9 |  |
| Eastern Yellow Wagtail | *Motacilla tschutschensis* | LC | 37 |  | 2 | 2 |
| Whimbrel | *Numenius phaeopus* | LC | 1 |  | 1 |  |
| Black-crowned Night-heron | *Nycticorax nycticorax* | LC | 49 | 34 | 455 | 39 |
| Black-naped Oriole | *Oriolus chinensis* | LC |  | 6 |  |  |
| Reed Parrotbill | *Paradoxornis heudei* | NT |  |  | 1 |  |
| Japanese Tit | *Parus minor* | LC | 61 | 21 | 16 | 14 |
| Eurasian Tree Sparrow | *Passer montanus* | LC | 2700 | 1132 | 4623 | 4386 |
| Common Pheasant | *Phasianus colchicus* | LC | 5 | 7 | 15 | 31 |
| Daurian Redstart | *Phoenicurus auroreus* | LC | 36 | 2 | 37 | 53 |
| Arctic Warbler | *Phylloscopus borealis* | LC | 1 |  | 3 |  |
| Dusky Warbler | *Phylloscopus fuscatus* | LC | 1 |  | 2 |  |
| Yellow-browed Warbler | *Phylloscopus inornatus* | LC | 12 |  | 4 |  |
| Pallas's Leaf Warbler | *Phylloscopus proregulus* | LC | 5 |  | 8 | 2 |
| Oriental Magpie | *Pica serica* | LC | 721 | 206 | 343 | 755 |
| Grey-capped Woodpecker | *Picoides canicapillus* | LC |  |  | 1 | 4 |
| Grey-faced Woodpecker | *Picus canus* | LC |  |  | 1 |  |
| Pacific Golden Plover | *Pluvialis fulva* | LC | 2 |  | 7 | 1 |
| Grey Plover | *Pluvialis squatarola* | LC |  |  | 2 |  |
| Plain Prinia | *Prinia inornata* | LC | 12 |  | 92 | 3 |
| Masked Laughingthrush | *Pterorhinus perspicillatus* | LC | 26 | 17 | 97 | 14 |
| Light-vented Bulbul | *Pycnonotus sinensis* | LC | 646 | 157 | 687 | 384 |
| Eastern Water Rail | *Rallus indicus* | LC |  |  | 1 |  |
| Chinese Penduline Tit | *Remiz consobrinus* | LC |  |  | 25 |  |
| Greater Painted-snipe | *Rostratula benghalensis* | LC |  |  | 1 |  |
| Stejneger's Stonechat | *Saxicola stejnegeri* | LC | 3 |  |  | 4 |
| Eurasian Woodcock | *Scolopax rusticola* | LC |  | 3 | 2 |  |
| Vinous-throated Parrotbill | *Sinosuthora webbiana* | LC | 156 | 24 | 164 | 132 |
| Spotted Dove | *Spilopelia chinensis* | LC | 315 | 281 | 836 | 406 |
| Eurasian Siskin | *Spinus spinus* | LC |  | 2 | 9 | 15 |
| Collared Finchbill | *Spizixos semitorques* | LC |  |  | 26 | 3 |
| White-cheeked Starling | *Spodiopsar cineraceus* | LC | 261 | 147 | 2043 | 179 |
| Red-billed Starling | *Spodiopsar sericeus* | LC | 8 | 13 | 88 | 7 |
| Oriental Turtle Dove | *Streptopelia orientalis* | LC | 34 | 274 | 158 | 32 |
| Red Turtle Dove | *Streptopelia tranquebarica* | LC | 23 | 7 |  |  |
| Common Starling | *Sturnus vulgaris* | LC |  |  | 3 |  |
| Little Grebe | *Tachybaptus ruficollis* | LC | 57 | 17 | 114 | 33 |
| Orange-flanked Bush-robin | *Tarsiger cyanurus* | LC | 9 |  | 2 | 1 |
| Spotted Redshank | *Tringa erythropus* | LC |  | 1 | 17 |  |
| Wood Sandpiper | *Tringa glareola* | LC |  |  |  | 25 |
| Common Greenshank | *Tringa nebularia* | LC | 1 | 1 | 3 |  |
| Green Sandpiper | *Tringa ochropus* | LC | 60 | 5 | 50 | 3 |
| Marsh Sandpiper | *Tringa stagnatilis* | LC | 1 |  | 1 |  |
| Dusky Thrush | *Turdus eunomus* | LC |  |  |  | 5 |
| Grey-backed Thrush | *Turdus hortulorum* | LC |  | 2 | 25 | 1 |
| Chinese Blackbird | *Turdus mandarinus* | LC | 415 | 213 | 220 | 447 |
| Naumann's Thrush | *Turdus naumanni* | LC | 178 | 1 | 7 |  |
| Eyebrowed Thrush | *Turdus obscurus* | LC | 1 |  | 7 |  |
| Pale Thrush | *Turdus pallidus* | LC |  |  | 1 | 1 |
| Yellow-legged Buttonquail | *Turnix tanki* | LC |  |  | 1 |  |
| Eurasian Hoopoe | *Upupa epops* | LC | 48 | 33 | 8 | 37 |
| Grey-headed Lapwing | *Vanellus cinereus* | LC | 2 | 11 | 251 | 7 |
| Northern Lapwing | *Vanellus vanellus* | NT | 40 | 40 | 17 | 9 |
| Brown Crake | *Zapornia akool* | LC | 1 |  | 14 |  |
| White's Thrush | *Zoothera aurea* | LC |  |  | 1 |  |

Notes: CZ: Changzhou Benniu International Airport; HA: Huai’an Lianshui International Airports; NJ: Nanjing Lukou International Airport; YT: Yangzhou Taizhou International Airport. VU: vulnerable species; NT: near threatened species; LC: least concern species.
